# Biclustering of gene expression data using reactive greedy randomized adaptive search procedure

**DOI:** 10.1186/1471-2105-10-S1-S27

**Published:** 2009-01-30

**Authors:** Smitha Dharan, Achuthsankar S Nair

**Affiliations:** 1 Centre for Bioinformatics, University of Kerala, Thiruvananthapuram, Kerala, 695 581, India; 2 Department of Computer Engineering, College of Engineering, Chengannur, Kerala, 689 121 India

## Abstract

**Background:**

Biclustering algorithms belong to a distinct class of clustering algorithms that perform simultaneous clustering of both rows and columns of the gene expression matrix and can be a very useful analysis tool when some genes have multiple functions and experimental conditions are diverse. Cheng and Church have introduced a measure called mean squared residue score to evaluate the quality of a bicluster and has become one of the most popular measures to search for biclusters. In this paper, we review basic concepts of the metaheuristics *Greedy Randomized Adaptive Search Procedure (GRASP)*-construction and local search phases and propose a new method which is a variant of *GRASP *called *Reactive Greedy Randomized Adaptive Search Procedure (Reactive GRASP) *to detect significant biclusters from large microarray datasets. The method has two major steps. First, high quality bicluster seeds are generated by means of k-means clustering. In the second step, these seeds are grown using the Reactive GRASP, in which the basic parameter that defines the restrictiveness of the candidate list is self-adjusted, depending on the quality of the solutions found previously.

**Results:**

We performed statistical and biological validations of the biclusters obtained and evaluated the method against the results of basic GRASP and as well as with the classic work of Cheng and Church. The experimental results indicate that the Reactive GRASP approach outperforms the basic GRASP algorithm and Cheng and Church approach.

**Conclusion:**

The Reactive GRASP approach for the detection of significant biclusters is robust and does not require calibration efforts.

## Background

Gene expression microarray is a highly popular technology that allows genome-wide measurement of RNA expression levels in a highly quantitative manner. Gene expression data is typically arranged as an *m *× *n *data matrix, with rows corresponding to genes and columns corresponding to experimental conditions. Conditions can be different environmental conditions or different time points corresponding to one or more environmental conditions. The (*m*, *n*)^th ^entry of the gene expression matrix represents the expression level of the gene corresponding to row *m *under the specific condition corresponding to column *n*. The numerical value of the entry is usually the logarithm of the relative amount of the mRNA of the gene under the specific condition. By analyzing the gene expression data, we can potentially determine which genes behave in similar ways, how genes interact, which genes contribute to the same pathway, and so on. The similarity between the genes can be analyzed by clustering the gene expression data. Cluster analysis plays an important role in the microarray data analysis through the grouping of genes into subsets with similar expression patterns or similar function. However, clustering has its limitations. Mainly, clustering works on the assumption that related genes behave similarly across all measured conditions. But a general understanding of cellular processes expects subsets of genes to be co-regulated and co-expressed only under certain experimental conditions, but behaves almost independently under other conditions [1]. In order to overcome the above shortcoming of clustering, the concept of biclustering is applied to gene expression data.

Biclustering was first described in the literature by Hartigan [2]. It refers to a distinct class of clustering algorithms that perform simultaneous row column clustering. Cheng and Church [3] were the first to apply it to gene expression data. Biclustering identifies subsets of genes and subsets of conditions that shares similar expression patterns, by performing simultaneous clustering of both rows and columns of the gene expression matrix. As a result, homogeneous sub matrices of the gene expression matrix are obtained and they are known as biclusters. In [3], Cheng and Church proposed a similarity score called *mean squared residue score *as a measure of coherence of the rows and columns in the bicluster. When all the elements in a bicluster are similar, the mean squared residue score is low. The lower the score, stronger the coherence exhibited by the bicluster, and better is the quality of the bicluster. However, from a biological point of view, the interest resides in biclusters with subset of genes showing similar behaviour and not with similar values. Hence the method aims at finding large and maximal biclusters with mean squared residue score below a certain threshold, δ, and the biclusters thus obtained are called δ-biclusters. The value of δ has to be estimated in advance, and it is different for every dataset [4].

In [3], Cheng and Church identified the problem of finding significant biclusters as being NP-Hard and employed a greedy node deletion algorithm in their search. Greedy search algorithms start with an initial solution and find a locally optimal solution by successive transformations that improve some cost function. The survey of biclustering algorithms for biological data analysis by Madeira and Oliveira [1] also identifies greedy search algorithms as a promising area. But greedy algorithms always make a choice that maximizes the local gain in the hope that this choice will lead to a globally good solution. It may make wrong decisions, gets stuck into local optima and thereby loose good biclusters. Metaheuristics technique such as Greedy Randomized Adaptive Search Procedure (GRASP) improves on pure greedy search due to their potential to escape from local minima.

In this work, we address the biclustering problem with a variant of the GRASP metaheuristics, Reactive GRASP, which is a combination of a semi-greedy heuristics and a local search procedure [5-7]. The approach starts from small high quality bicluster seeds, which are tightly co-regulated submatrices of the gene expression matrix. These seeds are further enlarged by adding more rows and columns to them. The seed generation phase is implemented using one-dimensional k-means clustering and the seeds are enlarged using the Reactive GRASP method. The algorithm makes use of *mean squared residue score *as the cost function to evaluate the quality of the obtained biclusters. To avoid getting stuck at local optima, Reactive GRASP is equipped with the heuristics for randomizing the search and thereby allows the search process to go beyond local optima. We evaluated our work against the classic work of Cheng and Church and also with our own work based on basic GRASP [8]. The results show that the Reactive GRASP method can generate larger and better biclusters than the others.

## Methods

### Model of a bicluster

A bicluster is defined on a gene expression matrix. A gene expression matrix is an *m *× *n *matrix, whose rows represents the genes, columns the experimental conditions and (i, j)^th ^element is a real number that represents the expression level of gene *i *under experimental condition *j*. Each row corresponds to the expression levels of a particular gene over all experimental conditions and each column corresponds to the expression levels of *m *genes under a specific experimental condition.

Let G = {g_1_, g_2 _..., g_m_} and C = {c_1_, c_2_,....., c_n_} represent a set of genes and a set of experimental conditions involved in a gene expression matrix, respectively. A bicluster is defined to be a subset of genes that exhibit similar behaviour under a subset of experimental conditions, and vice versa. Thus, in the gene expression matrix, a bicluster will appear as a sub matrix of it and represented as a pair A = (I, J) or simply as A_IJ_, where I ⊆ G and J ⊆ C. The rows and columns of the bicluster need not be contiguous as in the expression matrix.

A group of genes are said to be coherent if their level of expression reacts in parallel or correlates across a set of conditions. Similarly, a set of conditions may also have coherent levels of expression across a set of genes. The degree of coherence of a bicluster is measured using the concept of *mean squared residue score, HScore*, which represents the variance of a particular subset of genes under a particular subset of conditions with respect to the coherence. In [3], Cheng and Church defined the mean squared residue score as follows: It is defined for bicluster A_IJ _as the sum of the squared residues. R(a_ij_), the residue of an element a_ij _in the bicluster A_IJ_, i ∈ I and j ∈ J, is a measure of how well the element fits into that bicluster. It is defined to be:

(1) *R*(*a*_*ij*_) = *a*_*ij *_- *a*_*Ij *_- *a*_*iJ *_+ *a*_*Ij*_

Now, *HScore *is defined as in (2):

(2) $$\begin{array}{c} HScore=\frac{\sum_{i\in I,j\in J}{\left(R(a_{ij})\right)}^{2}}{\left|I\right|*\left|J\right|}\\ \text{where }a_{Ij}=\frac{\sum_{i\in I}a_{ij}}{\left|I\right|}\text{ is the column mean of column j}\\ a_{iJ}=\frac{\sum_{j\in J}a_{ij}}{\left|J\right|}\text{ is the row mean of row i}\\ a_{IJ}=\frac{\sum_{i\in I,j\in J}a_{ij}}{\left|I\right|*\left|J\right|}{\text{ is the mean of bicluster A}}_{\text{IJ}}. \end{array}$$

A bicluster is defined to be a δ-bicluster if *HScore *(*I*, *J*) ≤ δ, for some δ ≥ 0, where δ is the maximum acceptable *mean squared residue score*. The value of δ has to be estimated in advance, and it is different for every dataset [4]. The *HScore *gives an indication of how the data is correlated in the sub matrix – whether it has some coherence or is random. A matrix of equally spread random values over a range [a, b] has an expected *HScore *of (b-a)^2^/12 and it is independent of the size of the matrix. A high *HScore *indicates that the data is uncorrelated and a low *HScore *means that there is correlation in the matrix.

### Formulation of the biclustering problem

The biclustering problem is NP-hard as proven by Cheng and Church (2000) [3]. Thus no polynomial time algorithm exists and they might require exponential computation time in the worst-case. In this work, a heuristics based search method is adopted for finding the δ-biclusters in reasonable time. The problem is formulated as an optimization problem, which aims at minimizing the *HScore*. The algorithm has two major phases. In the first phase, an initial set of tightly co-regulated seed biclusters are generated. The second is the heuristics based seed growing phase, in which the seeds are further refined and enlarged by adding more genes and conditions until their *HScore *reaches a certain predetermined threshold.

Generally pure greedy algorithms make good local choices in the hope that they result in an optimal solution. But in case of situations like local minima, ridges and plateaus, they may make wrong decisions and thereby loose optimal biclusters [9,10]. It is thus unlikely that a global maximum or maximal δ-bicluster will be found. Metaheuristic algorithms incorporate mechanisms to prevent getting trapped in such confined areas of the search space. So here we use the Reactive Greedy Randomized Adaptive Search procedure (Reactive GRASP), which is a variant of the GRASP metaheuristics is used for the extraction of δ-biclusters.

A good seed of a possible bicluster is actually a small bicluster whose *HScore *has reached the requirement but the volume may not be maximal. The simplest ways to generate quality bicluster seeds is to perform standard one-dimensional clustering on both rows and columns of the expression matrix separately and then combine them to generate different seeds [9,11]. We have used k-means algorithm with cosine angle distance as the distance measure.

During seed generation, the gene expression matrix is partitioned into *p *gene clusters and *q *condition clusters. As the number of genes per each gene cluster is too high, each gene cluster is further divided into subclusters. As a result we get *x *geneclusters and *q *condition clusters. By combining these gene and condition clusters, we get x_*_q disjoint submatrices. Finally, the *HScore *values of the entire x_*_q submatrices are calculated and those having *HScore *value less than a threshold are selected as the seeds.

### Greedy randomized adaptive search procedure: a review

GRASP is implemented as a multistart procedure, where each iteration is made up of a construction phase, where a randomized greedy solution is constructed, and a local search phase. The local search phase starts at the constructed solution and applies iterative improvement until a locally optimal solution is found. The GRASP algorithm is summarized in Table 1.

**Table 1:** Algorithm of greedy randomized search procedure

Algorithm GRASP (Seed)	
Current = Seed;	
While <termination condition not met> do	
Solution ← Greedy_Randomized_ Construction (Current);	
Solution ← Local_Search (Solution);	
Current ← Solution;	
End	

During the construction phase, the set of candidate elements are formed by all elements that can be incorporated to the partial solution under construction without destroying feasibility. The quality of a candidate element is determined by its contribution, at that point, to the cost of the solution being constructed. A greedy evaluation function measures this contribution for each candidate element. Accordingly a restricted candidate list (RCL) is constructed of high quality candidate elements. The number of elements in RCL can be limited either by rank or by quality relative to other candidates. In rank based RCL, *r *candidates with smallest greedy function value are selected from the candidate list and *r *determines how greedy or random the construction will be. The quality based RCL uses a greedy function cutoff value and only considers candidates with a greedy function value no greater than the cutoff. To implement this, a real-valued RCL parameter α ∈ [0..1] is used and it determines which elements are to be placed in the RCL at each iteration of the construction phase. Since in the case of a minimization problem, the case α = 0 corresponds to a pure greedy algorithm, while α = 1 is equivalent to a pure random construction. Thus, the parameter 'α' controls the amount of greediness and randomness in the algorithm. In basic GRASP implementation, the same α is used along all iterations and it is usually determined through experimentation. In our earlier work based on GRASP, we used a quality-based implementation of RCL for the extraction of biclusters [8]. The element to be incorporated into the partial solution is randomly selected from those in the RCL. Once the selected element is incorporated to the partial solution, the candidate list is updated and the incremental costs are re-evaluated. The description of the construction phase is given in Table 2.

**Table 2:** Algorithm of greedy randomized construction phase

Algorithm Greedy_Randomized_Construction(Seed)	
Solution ← Seed	
Calculate the incremental costs of the elements not included in the current solution	
Build the candidate list	
While <termination condition not met> do	
Build the restricted candidate list (RCL)	
Select an element s at random from RCL	
Solution ← Solution ∪ s	
Recalculate the incremental costs and candidate list	
End	
Return Solution	
End	

The solution generated by a greedy randomized construction is not necessarily optimal. The local search phase usually improves the constructed solution. A local search algorithm works in an iterative fashion by successively replacing the current solution by a better solution in the neighbourhood of the current solution. It terminates when no better solution is found in the neighbourhood. In [6], Resende and Ribeiro reported that in most cases both first-improving and best-improving local search strategies lead to the same final solution, but in case of smaller computation times, the former outperforms the latter. Hence in our algorithm, a first improving strategy is used for the implementation of the local search. The description of the algorithm is given in Table 3. In the algorithm *N *(solution) represents the neighbourhood of the solution and *f *represents the cost function corresponding to the problem.

**Table 3:** Algorithm of local search phase

Algorithm Local_Search (Solution)	
While there exists *s *∈ *N *(Solution) such that *f *(Solution ∪ *s*) <*f *(Solution) do	
Solution ← Solution ∪ *s*	
End	
Return Solution	

An especially appealing characteristic of GRASP is the ease with which it can be implemented. Few parameters need to be set and tuned. Basic implementation of GRASP relies exclusively on two parameters. The first controls the number of construction/local search iterations that will be applied and the second, the RCL parameter α, controls the blend of randomness and greediness in the solution construction procedure. But in [12], Prais and Ribeiro showed that using a single fixed value for α often hinders finding a high quality solution, which eventually could be found if another value was used. They proposed an extension of the basic GRASP called Reactive GRASP, in which the parameter α is not fixed, but instead is selected at each iteration from a discrete set of possible values. The solution values used along the previous iterations serve as a guide for the selection process. Reactive GRASP includes a memory mechanism that enables good solutions found in earlier iterations of the search to influence the search later.

### Biclustering using GRASP

Biclustering using GRASP is implemented as a two-step procedure. In the first, the GRASP iterations are performed over the seed bicluster to enlarge it column wise and during the second step, another set of GRASP iterations add more rows while keeping the *HScore *below a certain predetermined threshold. During the construction phase of each GRASP iteration, a restricted candidate list (RCL) is made from the candidate list according to the greedy evaluation function as shown in (3).

(3) RCL ← {s ∈ C or G|HScore (Solution ∪ s) ≤ Smin + α (Smax-Smin)}

where

Smin ← min {HScore (Solution ∪ t)|∀ t ∈ C or G}

Smax ← max {HScore (Solution ∪ t)|∀ t ∈ C or G}

C is the candidate list of conditions

G is the candidate list of genes

α is the RCL threshold parameter, α ∈ [0..1]

The element to be incorporated into the partial solution is randomly selected from those in the RCL. Once the selected element is incorporated to the partial solution, the candidate list is updated and the incremental costs are reevaluated. The quality of elements in the RCL depends greatly on the threshold parameter α. α = 1 corresponds to pure random construction, as α → 0; the algorithm behaves more or less like greedy algorithms. Hence for better results, proper tuning on the value of α is required and usually it is determined through experimentation. Since the solution constructed during the construction phase is not necessarily optimal, the local search is performed over the constructed solution for further improvements.

### Biclustering using Reactive GRASP

In Reactive GRASP, instead of using a fixed value for the parameter α, it uses different values in different iterations. During each iteration the value of α is selected from a discrete set of possible values, say R = {α_1_,.....,α_m_}. The solution values found along the previous iterations serve as a guide for the selection process. Let p_i _be the selection probability associated with the choice of α_i_, for i = 1,....., m. Initially all p_i_'s are made equal to 1/m. The selection probabilities are periodically reevaluated using information collected during the search. After each iteration, with a particular α_i_, the difference in the current solution value and the value of the solution obtained in the previous iteration is calculated. Let A_i _be the average value of such differences obtained taking α = α_i _in the construction phase. The probability distribution is updated after each GRASP iteration by taking

(4) $$p_{i}=\frac{q_{i}}{\sum_{j=1}^{m}q_{j}}$$

with q_i _= 1/A_i _for i = 1,..., m. The value of q_i _will be larger for values of α = α_i _leading to the best solutions on the average. Larger values of q_i _correspond to more suitable values for the parameter α. The probabilities associated with these more appropriate values will then increase when they are reevaluated.

### Significance evaluation

The statistical significance of the biclusters obtained is evaluated by calculating the *p-values*, which signify how well they match with the known gene annotation. A smaller *p-value*, close to zero, is indicative of a better match [13]. Tanay et al. [14] proposed a technique called *correspondence plot *to evaluate the biclusters using prior biological knowledge. It takes advantage of a known classification of genes or experimental conditions. The plot depicts the distribution of *p-values *of the biclusters produced based on a known classification of conditions or a given gene annotation. For each value of *p *on a logarithmic scale, the plot presents the fraction of biclusters whose *p-value *is at most *p *out of the, say *b*, best biclusters. The *p-values *of the biclusters are calculated according to the known classification of genes as follows: It is the probability of finding at least *k* genes in a bicluster of *n* genes, belonging to a specific functional category comprising *f* genes out of total *g* annotated genes is given by

(5) $$p_{i}=\sum_{i=k}^{\min(n,f)}\frac{\left({\;}^{f}C_{i})({\;}^{g-f}C_{n-i}\right)}{{\;}^{g}C_{n}}$$

In the correspondence plot, early departure of the curve from the x-axis of the plot indicates the existence of biclusters with low *p-values*. Consequently, area under the curve shows the approximate degree of statistical significance of the biclusters used to draw the curve [15]. For statistical validation, we used the 30 known categories of Yeast genes reported by Tavazoie et al. [16]. They used an iterative optimization-based partitional clustering to group 3000 genes into 30 expression classes which were highly enriched for genes of similar function on time-series of mRNA abundance, measured over two synchronized Saccharomyces cerevisiae cell cycles. Also to evaluate the biological significance of the obtained biclusters, in terms of the associated biological processes, molecular functions and cellular components respectively, we have used the SGD GO gene ontology term finder [17].

### Time complexity of the algorithm

To find one bicluster from a seed, the algorithm has to compute the *HScores *of all the submatrices that may result from any row (gene) or column (condition) addition, before each choice can be made. Since *m *and *n *are the total number of genes and conditions in the gene expression matrix, the *HScore *can be calculated in O (*mn*) time. Hence in the worst case, the algorithm requires O (*mn *(*m+n*)) time.

## Results

### Dataset used

The proposed biclustering algorithm is implemented in *Matlab *and tested on the *Yeast Saccharomyces Cerevisiae *cell cycle expression dataset. The dataset is based on Tavazoie et al. [16] and is taken from [18]. It is a collection of 2884 genes and 17 experimental conditions (time points), having 34 null entries with -1 indicating the missing values. All entries are integers lying in the range 0 to 595. The value of δ is used as an upper limit of allowable dissimilarity among genes and conditions. A higher δ is indicative of diminishing homogeneity. Hence in our approach we used a value of 200 for δ.

### Biclustering using GRASP and Reactive GRASP

The Reactive GRASP and basic GRASP algorithms begin the search from tightly co-regulated bicluster seeds. These seeds are enlarged by adding more genes and conditions until the *HScore *of the bicluster reaches the given threshold (δ) value. The seed growing is implemented in two phases-construction and local search. During the construction phase it picks a random move from among a restricted candidate list (RCL) of possible best moves. In case of a row or a column addition, RCL contains only top quality rows or columns whose inclusion to the current partial solution doesn't affect feasibility of the solution. The quality of elements in the RCL depends greatly on the threshold parameter α and in GRASP, the same value for α is used along all iterations. But it often hinders finding a high quality solution, which could be found if another value was used. Hence in reactive GRASP a self-adjustable α is used. During each iteration, the value of α is selected from a discrete set of possible values depending on the selection probabilities. The elements of the discrete set can be in the range [0..1]. In order to ensure the quality of solutions, we have completely eliminated the extreme values. Hence in our implementation of Reactive GRASP, we have used 10 numbers in the range [0.25 .. 0.60]. After the construction phase, local search is performed on these solutions to further improvise them.

**Figure 1:**
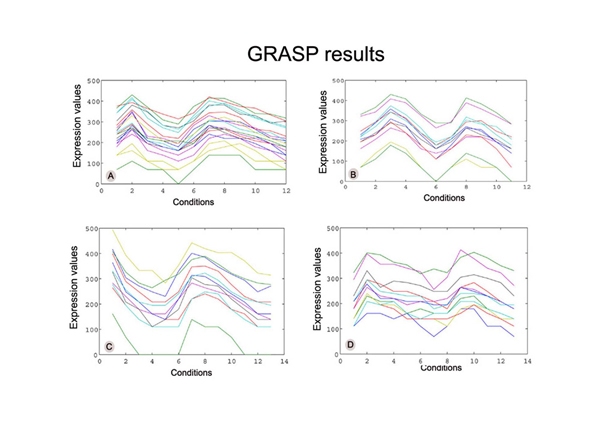
**Biclusters extracted from the Yeast gene expression data using GRASP**. The biclusters are labelled as A, B, C and D. The number of genes, number of conditions, HScore, p-value of the biclusters are (A) (20, 12, 198.70, 2.8479e-020) (B) (12, 11, 197.7, 3.6879e-015) (C) (11, 13, 197.79, 6.0545e-014) (D) (12, 13, 199.9, 2.9778e-009).

**Figure 2:**
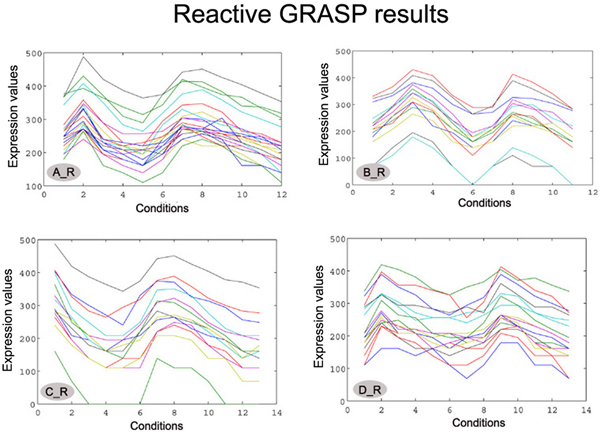
**Biclusters extracted from the Yeast gene expression data using Reactive GRASP**. The biclusters are labelled as A_R, B_R, C_R and D_R. The number of genes, number of conditions, HScore, p-value of the biclusters are (A_R) (22, 12, 198.81, 6.9185e-025) (B_R) (14, 11, 196.08, 1.3464e-017) (C_R) (14, 13, 197.63, 1.3464e-017) (D_R) (17, 13, 199.02, 7.7195e-019).

In Figure 1, four of the biclusters found by the GRASP algorithm on the Yeast dataset are shown. From a visual inspection of the biclusters presented, one can notice that the genes present a similar behaviour under a set of conditions only. Figure 2 shows the set of biclusters derived from the same seeds using the Reactive GRASP. The *p-values *of the biclusters show significant change from that of the GRASP method.

### Statistical and biological significance evaluation

**Figure 3:**
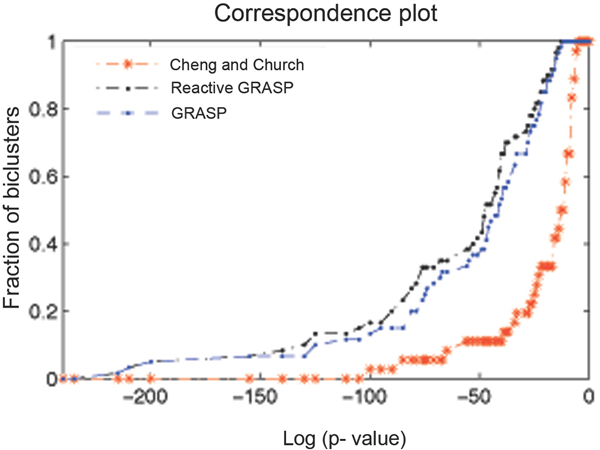
Correspondence plot for the Yeast dataset.

The statistical significance of the biclusters obtained is evaluated by calculating the *p-values*, which signify how well they match with the known gene annotation. For a statistical comparison of the biclusters produced by our method with that of Cheng and Church, we used the *correspondence plot *proposed by Tanay et al. [14]. Figure 3 presents the correspondence plot. In the plot, early departure of the curve from the x-axis of the plot indicates the existence of biclusters with low *p-values*. Consequently, area under the curve shows the approximate degree of statistical significance of the biclusters used to draw the curve [15]. It shows that the biclusters generated by the Reactive GRASP algorithm tend to be more statistically significant than the basic GRASP and Cheng and Church approach. While plotting the correspondence plot, we choose those biclusters in which more than 60% of their annotated members had the same class. Out of those, we only used biclusters that were functionally enriched.

**Figure 4:**
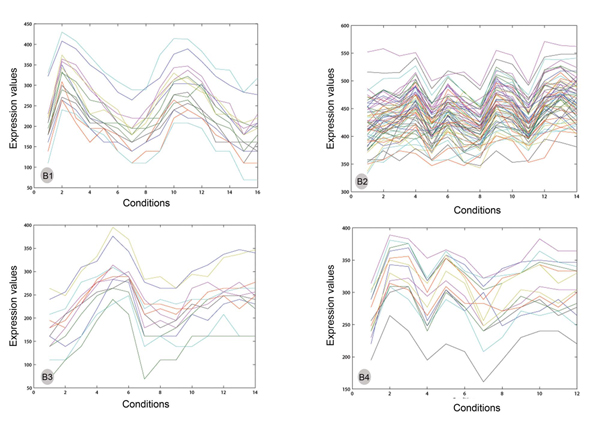
**Sample biclusters obtained from Reactive GRASP**. The biclusters are labelled as B1, B2, B3 and B4. The number of genes, number of conditions, HScore are (B1) (16, 16, 199.79) (B2) (61, 14, 143.03) (B3) (12, 14, 198.50 (B4) (14, 12, 125.83).

We apply the yeast genome gene ontology termfinder [17] on each discovered biclusters to evaluate their biological significance in terms of associated biological processes, molecular functions and cellular components respectively. For a sample set of biclusters in Figure 4, Table 4 describes the top GO terms of the three categories with the lowest p-values. The GO terms are displayed in the decreasing order of significance. For the bicluster labelled B1, the genes *RFA1, POL12, POL30, CDC9, MSH6, RAD27, CDC45, RFA2*, and *CDC21 *are together involved in the process of DNA-dependent DNA replication, DNA replication and DNA metabolic process. Each GO term is associated with a tuple, for example DNA-dependent DNA replication (9, 1.40e-11) indicates 9 out of the total 16 genes of B1 belong to this process and their statistical significance is 1.40e-11 i.e. p-value. Also from the table it is clear that the biclusters extracted are distinct along each category. Existence of biclusters comprising a significant proportion of those genes that are considered similar biologically is proof that a specific biclustering technique produces biologically relevant results. This shows that our algorithm is capable of identifying a broader range of biologically significant biclusters.

**Table 4:** Top GO terms (process, function, component) of the biclusters in Figure 4.

Bicluster	Process	Function	Component
B1	DNA-dependent DNA replication (9, 1.40e-11), DNA replication (9, 3.48e-10), DNA metabolic process(11, 5.45e-09), DNA repair (8, 4.20e-07)	double-stranded DNA binding (4, 4.09e-06), structure-specific DNA binding (4, 8.45e-05), sequence-specific DNA binding(4, 4.5e-04)	replication fork (7,4.10e-11), chromosomal part(8, 2.77e-07) Chromosome(8, 7.84e-07)

B2	Translation (32,2.87e-15), Cellular protein metabolic process (36, 3.59e-9), Protein metabolic process(36, 4.81e-9), Cellular macromolecule biosynthetic process(36,2.51e-08)	Structural constituent of ribosome (28, 3.59e-25) Structural molecule activity (28, 4.71e-20) Translation elongation factor activity (3, .00213)	Cytosolic ribosome (29, 8.7e-30), Cytosolic part (29, 1.2e-26) Ribosome(29, 1.09e-25), Ribosomal subunit(32, 1.13e-24), Ribonucleoprotein complex(34, 7.48e-18) Cytosol (33, 2.05e-17)

B3	Cell cycle process (6, 0.00055), Cell cycle(6, 0.00109), Mitotic cell cycle(5, 0.00145), Cell cycle phase(5, 0.00505)	Kinase regular activity (2, 0.00410)	Cellular bud (6, 1.73e-06), Site of polarized growth(6, 1.87e-06) Cellular bud neck(5, 2.22e-05) Incipient cellular bud site(3,0.00129)

B4	Ribonucleoprotein complex biogenesis and assembly (10, 2.08e-07) Ribosome biogenesis(9, 1.07e-06)	Methyl transferase activity (3,8.56e-03) Transferase activity, transferring one-carbon groups(3,9.17e-03)	Organelle lumen(9,1.2e-04) Intracellular organelle lumen(9, 1.2e-04) Nuclear lumen(8, 2.2e-04) Nucleolus(6, 5.9e-04)

## Discussion

In GRASP, an appropriate value of the RCL parameter α is clearly critical and relevant to achieve a good balance between computation time and solution quality. But it requires a lot of experimentation overhead to fix the value. Hence we proposed Reactive GRASP, which is a variant of GRASP, to tackle the biclustering problem. Reactive GRASP, being self-adjustable, changes the value of the RCL parameter periodically according with the quality of the solutions obtained recently. The approach looks more robust and doesn't require calibration efforts. The experimental results also indicate that the Reactive GRASP approach outperforms the basic GRASP and Cheng and Church algorithm.

## Conclusion

This paper dealt with the extraction of biclusters in microarray gene expression data. We addressed the problem with a heuristics based seed-growing algorithm – the Reactive GRASP metaheuristics – which is a variant of the GRASP approach. The seed biclusters, which are tightly co-regulated submatrices, are obtained by performing k-means clustering algorithm to the rows and columns separately and then by combining them. During seed growing, these seed biclusters are further refined by adding more rows and columns to extend their size while keeping the mean squared residue score below a certain predefined threshold. Since Reactive GRASP being semi-greedy, it tries to combine the advantages of both random and greedy solution constructions and thereby gives the possibility to escape from locally optimal solutions. Also it makes use of a memory mechanism that enables good solutions found in earlier iterations of the search to influence the search later. To our knowledge, biclustering using GRASP techniques has not till been reported before in the literature. We have conducted and tested our algorithm on the Yeast dataset. The experimental results show that the algorithm is successful in finding statistically and biologically verifiable biclusters. Also the correspondence plot reveals that the algorithm finds biclusters that better aligned more closely with prior biological knowledge than that of basic GRASP and Cheng and Church approach.

## Competing interests

The authors declare that they have no competing interests.

## Authors' contributions

SD and ASN participated in the method design. SD performed all the experiments. SD and ASN participated in the result discussion. SD and ASN participated in the paper writing. ASN finalized the submission. All authors read and approved the final manuscript.
